# Shedding “LIGHT” on the Link between Bone and Fat in Obese Children and Adolescents

**DOI:** 10.3390/ijms21134739

**Published:** 2020-07-03

**Authors:** Giacomina Brunetti, Maria Felicia Faienza, Laura Piacente, Giuseppina Storlino, Angela Oranger, Gabriele D’Amato, Gianpaolo De Filippo, Silvia Colucci, Maria Grano

**Affiliations:** 1 Department of Basic and Medical Sciences, Neurosciences and Sense Organs, Section of Human Anatomy and Histology, University of Bari, 70124 Bari, Italy; silviaconcetta.colucci@uniba.it; 2 Department of Biomedical Science and Human Oncology, Paediatric Unit, University of Bari, 70100 Bari, Italy; mariafelicia.faienza@uniba.it (M.F.F.); laura.piacente@uniba.it (L.P.); 3 Department of Emergency and Organ Transplantation, Section of Human Anatomy and Histology, University of Bari, 70124 Bari, Italy; g.storlino@gmail.com (G.S.); angelaoranger@yahoo.it (A.O.); maria.grano@uniba.it (M.G.); 4 Neonatal Intensive Care Unit, Di Venere Hospital, 70131 Bari, Italy; gab59it@yahoo.it; 5 Assistance Publique-Hôpitaux de Paris, Hôpital Robert Debré, Service d’Endocrinologie Diabétologie Pédiatrique, 75019 Paris, France; gianpaolo.defilippo@aphp.fr

**Keywords:** LIGHT/TNFSF14, obese subjects, osteoclasts, bone mineral density

## Abstract

Obesity may affect bone health, but literature reports are contradictory about the correlation of body mass index (BMI) and bone markers. LIGHT, one of the immunostimulatory cytokines regulating the homeostasis of bone and adipose tissue, could be involved in obesity. The study involved 111 obese subjects (12.21 ± 3.71 years) and 45 controls. Patients underwent the evaluation of bone status by quantitative ultrasonography (QUS). LIGHT amounts were evaluated in sera by ELISA, whereas its expression on peripheral blood cells was evaluated by flow cytometry. Osteoclastogenesis was performed by culturing peripheral blood mononuclear cells (PBMCs) with or without anti-LIGHT antibodies. Obese patients showed significant high BMI-standard deviation score (SDS), weight-SDS, and Homeostatic model assessment for insulin resistance (HOMA-IR) that negatively correlated with the reduced Amplitude Dependent Speed of Sound (AD-SoS)-Z-score and Bone Transmission Time (BTT-Z)-score. They displayed significantly higher serum levels of LIGHT compared with controls (497.30 ± 363.45 pg/mL vs. 186.06 ± 101.41 pg/mL, *p* < 0.001). LIGHT expression on monocytes, CD3+-T-cells, and neutrophils was also higher in obese patients than in the controls. Finally, in PBMC cultures, the addition of anti-LIGHT antibodies induced a significant osteoclastogenesis inhibition. Our study highlighted the high serum levels of LIGHT in obese children and adolescents, and its relationship with both the grade of obesity and bone impairment.

## 1. Introduction

There are numerous evidences showing that disorders with altered bone development, particularly during growth, may affect bone health in adulthood [1]. It is also known that obese children have low bone mineral density and a higher risk of osteoporosis and fractures than normal weight subjects [2], but there are conflicting data about the correlation between body mass index (BMI) and markers of bone remodeling [3,4,5]. However, the cellular and molecular mechanisms by which obesity and bone remodeling can mutually influence each other remains to be elucidated and deepened. Obese children, whose numbers increase in almost all countries, have a fivefold increased risk to remain obese during adulthood and to develop non-communicable diseases (NCDs) at a younger age, like cardiovascular diseases [6,7], different types of cancer, and metabolic and musculoskeletal disorders [8,9,10,11,12]. Obesity is characterized by low-grade chronic inflammation, with anomalous cytokine production and activation of inflammation signaling pathways, leading to the development of obesity-related disorders [13]. Inflammation and in general immune cell activity also may affect bone cell activity and thus bone remodeling [14]. Consequently, the cross-talk between adipose tissue, inflammatory, and metabolic pathways [15] may also influence endochondral longitudinal bone growth together with alteration of nutrients, minerals, and hormone metabolism [16]. In obese subjects, the changed amounts of some molecules such as Tumor Necrosis Factor α (TNFα), sclerostin, Dickkopf-1 (DKK1), serotonin, Interleukin-6 (IL-6), and advanced glycation end products (AGEs) may negatively affect osteoblastogenesis, with consequent impairment of bone formation (revised by Roy et al. [17]). In parallel, many pro-inflammatory cytokines involved in obesity are also crucial mediators of osteoclastogenesis, with consequent increase of bone resorption activity [18], and thus supporting the link between obesity and altered bone turnover. One of these cytokines could be represented by LIGHT (homologous to Lymphotoxins exhibiting Inducible expression and competing with herpes simplex virus Glycoprotein D for herpes virus entry mediator (HVEM), a receptor expressed by T lymphocytes) [19,20,21,22,23,24,25,26]. LIGHT/TNFSF14 is member of TNF superfamily and it is a key cytokine of the TNF-lymphotoxin network [27,28,29], expressed by immune cells, with a key role as a co-stimulatory molecule [30]. Interestingly, studies showed elevated LIGHT levels in obese adults compared to controls [26].

Numerous authors evaluated the role of LIGHT in adipogenesis obtaining conflicting results. In detail, Tiller et al. reported that LIGHT inhibits the differentiation of adipocytes without altering their metabolism [31]. Otherwise, Kim et al. showed that LIGHT has a crucial part in the inflammatory responses of adipose tissue by enhancing macrophage/T-cell infiltration as well as inflammatory cytokine expression using murine cell lines and an in vivo model [32]. Liu et al. showed that LIGHT supports the adipogenic differentiation of human mesenchymal stem cells in vitro [33]. Furthermore, Kou et al. demonstrated that LIGHT signaling reduces beige fat biogenesis in murine models [34]. However, there are no data in the literature evaluating the levels of LIGHT in the sera and on circulating cells of obese children and adolescents.

Thus, based on literature data, this study was designed to evaluate the serum levels of LIGHT and its correlation with bone metabolism markers and quantitative ultrasound measurements of bone quality in a group of children and adolescents with different grades of obesity.

## 2. Results

### 2.1. The Grade of Obesity Is Related to Bone Status in Obese Subjects

Clinical characteristics, lipid metabolism, glycemic control, bone metabolism markers, and parameters of bone quality of patients and controls are reported in Table 1. Obese patients showed significantly higher BMI- standard deviation score (SDS) weight-SDS, waist circumference, levels of insulin and Homeostatic model assessment for insulin resistance (HOMA-IR) than controls (*p* < 0.001). 

In obese patients the averages of Amplitude Dependent Speed of Sound (Ad-SoS)-Z-score and Bone Transmission Time (BTT)-Z-score were within the normal range but significantly reduced in respect to controls (*p* < 0.05). The patients also displayed significantly high levels of parathyroid hormone (PTH) (*p* < 0.05), whereas 25(OH) vitamin D levels were reduced compared to the controls, but the difference did not reach the statistical significance. Furthermore, we observed the significant correlation of BMI-SDS, weight-SDS, HOMA-IR and quantitative insulin sensitivity check index (QUICKI) with bone metabolism markers and bone quality parameters (Table 2). 

### 2.2. Obese Patients Display Increased LIGHT Serum Levels

**Figure 1:**
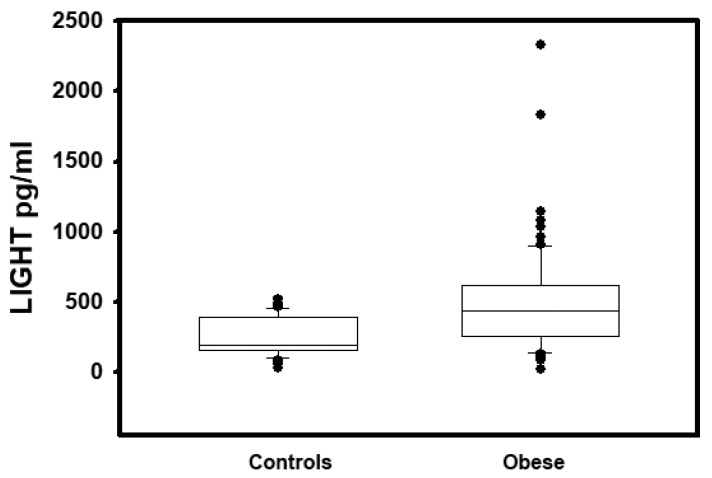
High levels of LIGHT in obese subjects. In controls and all obese subjects, the serum levels of LIGHT were measured by ELISA. Significant increased levels of LIGHT (497.30 ± 363.45 pg/mL vs. 186.06 ± 101.41 pg/mL *p* < 0.001) were found in obese patients, with respect to controls.

To understand if differences in circulating LIGHT levels were related to bone impairment in obese patients, we investigated LIGHT serum concentration and its correlations with different bone and metabolic parameters. Significant increased levels of LIGHT (497.30 ± 363.45 pg/mL vs. 186.06 ± 101.41 pg/mL, *p* < 0.001, Figure 1) were found in obese patients, with respect to controls. Interestingly, the high LIGHT levels in obese subjects correlated both with BMI and bone quality parameters. In detail, the cytokine levels positively correlated with age (*r* = 0.29, *p* < 0.03), Tanner stage (*r* = 0.207, *p* < 0.05), weight-SDS (*r* = 0.448, *p* < 0.0001), and BMI-SDS (*r* = 0.416, *p* < 0.0002), conversely a negative correlation with Ad-Sos-Z-score (*r*= −0.445, *p* < 0.02) and BTT-Z-score (*r* = −0.341, *p* < 0.05) was found. With adjustment for age, LIGHT levels also correlated positively with total cholesterol (*r* = 0.112, *p* < 0.001), LDL (*r* = 0.206, *p* < 0.0001), and triglycerides (*r* = 0.242, *p* < 0.0001), and negatively with HDL (*r* = −0.144, *p* < 0.0001), 25(OH) vitamin D (*r* = −0.173, *p* < 0.0001), and osteocalcin (*r* = −0.232, *p* < 0.0001). 

### 2.3. Circulating Monocytes, T-Cells and Neutrophils of Obese Patients Show High Levels of LIGHT

**Figure 2:**
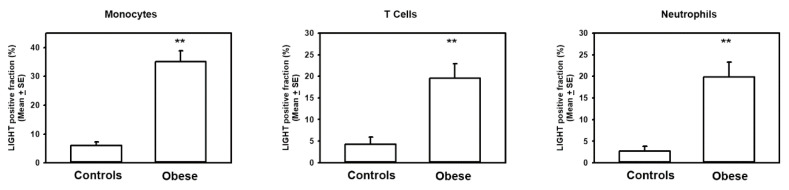
High expression of LIGHT in obese subjects. Histograms represent the percentage of LIGHT expression on CD14+ monocytes, CD3+ T cells, and neutrophils of controls and obese subjects, evaluated by flow cytometry. Statistics: *T*-test, *n* = 40; ** *p* < 0.001.

It is known that LIGHT is expressed on immune cells, thus we evaluated LIGHT expression on circulating monocytes, T cells, and neutrophils by flow cytometry. We found a significant increase of the percentage of LIGHT+ monocytes, CD3+ T cells, and neutrophils in obese subjects compared to the controls (Figure 2). With adjustment for age, in obese patients we assessed a positive correlation of LIGHT expression on monocytes with Tanner stage (*r* = 0.133, *p* < 0.01), glucose levels (*r* = 0.209, *p* < 0.0001), and BTT-Z-score (*r* = 0.592, *p* < 0.0001), whereas a negative correlation with alkaline phosphatase (*r* = −0.266, *p* < 0.0001) and 25(OH) vitamin D (*r* = −0.145, *p* < 0.009) was determined.

### 2.4. LIGHT Mediates Osteoclastogenesis on PBMC of Obese Patients

**Figure 3:**
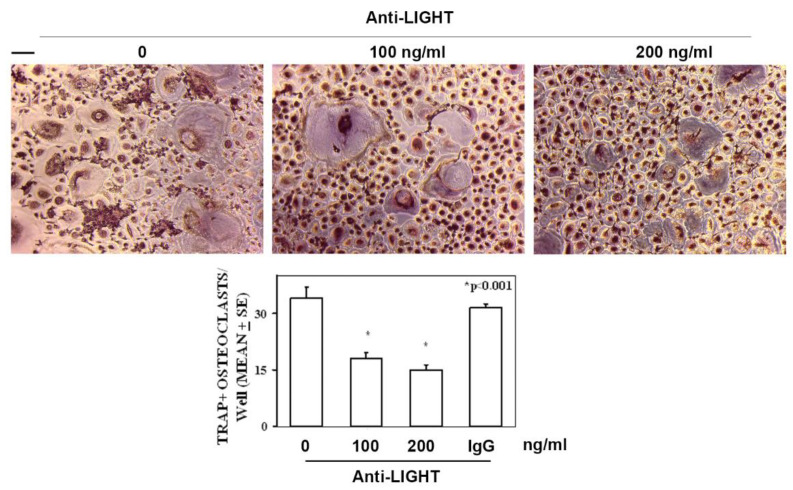
Anti-LIGHT mAb inhibits the osteoclast formation in cultures from obese subjects. Representative images of multinucleated tartrate-resistant acid phosphatase-stained (TRAP+) osteoclasts differentiated from peripheral blood mononuclear cell (PBMC) cultures of obese subjects, cultured in the presence of anti-LIGHT mAb or control anti-IgG mAb. The number of multinucleated and TRAP+ cells, identified as osteoclasts, are represented in the graphs as mean ± SE * *p* < 0.001. Statistics: *T*-test, *n* = 30. Scale bar 100 μm.

Based on our previous study demonstrating that spontaneous osteoclastogenesis occurred in peripheral blood mononuclear cells (PBMCs) of obese patients without the addition of macrophage colony-stimulating factor (MCSF) and Receptor activator of nuclear factor kappa-Β ligand (RANKL), otherwise necessary in PBMCs of normal subjects, [35], here we investigated the effect of LIGHT on this process. With this purpose, an anti-LIGHT antibody, at different concentrations, was used on the PBMC cultures of obese subjects to evaluate osteoclastogenesis. We demonstrated that the increasing doses of anti-LIGHT antibodies induced a dose-dependent reduction of osteoclast number in PBMCs from obese patients, supporting the involvement of LIGHT in the spontaneous osteoclast formation (Figure 3).

### 2.5. Bone and Metabolic Parameters Are the Most Important Predictors of LIGHT Levels

By multiple linear regression analysis, we explored the factors associated with the high LIGHT serum levels in obese subjects (Table 3). With adjustment for age, multiple linear regression analyses for LIGHT, as the dependent variable, showed that Tanner stage, weight-SDS, BMI-SDS, total cholesterol, 25(OH) vitamin D, osteocalcin, AD-SoS-Z-score, and BTT-Z-score are the most important predictors (*r* = 0.768, *p* < 0.0001).

## 3. Discussion

In the present study performed on a pediatric population of obese subjects, we demonstrated important findings: (1) the strong correlation of BMI-SDS, weight-SDS, HOMA-IR and QUICKI with bone metabolism markers and bone quality parameters; (2) high levels of LIGHT in the sera and on circulating monocytes, T-cells, and neutrophils; (3) the involvement of cytokine in the spontaneous osteoclastogenesis, *in vitro*, in PBMCs of obese patients.

Consistent with the strict correlation that we found between the grade of obesity (BMI-SDS, weight-SDS) and bone markers and quality (i.e., 25(OH) vitamin D, BTT-Z-score), several studies evaluated the impact of obesity on bone remodeling. In detail, in a cross-sectional study involving 60 females (range 10–19 years of age), Weiler et al. found that body fat percentage was related to the suboptimal achievement of peak bone mass [36]. Furthermore, Goulding et al. showed that elevated adiposity is linked with increased risk of distal forearm fractures in male children and adolescents [37]. Very recently, Geserick et al. reported that BMI was found to affect bone markers in children and adolescents with obesity [38]. In detail, age- and gender-adjusted bone formation and bone resorption markers were found to be significantly lower in obese children compared to the controls. Studies about the correlation of BMI and the bone markers Procollagen type 1 N-pro-peptides (PINP) and carboxy-terminal cross-linking telopeptide of type 1 collagen (CTX-I) are rare and controversial: some studies describe an increase [39,40], but others show a reduction of these bone markers in obese children [5,41]. Moreover, Geserick et al. also reported that obese children have significantly lower 25(OH) vitamin D and increased PTH-SDS levels compared to the controls [38]. Consistently, we found increased levels of PTH in our patients, whereas 25(OH) vitamin D levels, although lower compared with the controls, did not reach the statistical significance. 

Our data also showed the negative correlation between QUS parameters and HOMA-IR. QUS parameters were also related to QUICKI. Several indicators are used to assess insulin sensitivity/resistance. The HOMA-IR index indicates insulin resistance based on the amount of fasting plasma glucose and insulin. Several studies suggest that QUICKI can be a good alternative for HOMA-IR [42]. Moreover, a recent study showed a significant correlation between anthropometric and cardiometabolic parameters with both HOMA-IR and QUICKI [42].

In our study, we found a significant correlation between BMI-SDS and both HOMA-IR and QUICKI with bone metabolism markers and bone quality parameters, thus demonstrating that these two indices of insulin resistance are strongly related with the severity of obesity and to bone impairment.

Interestingly, we also demonstrated the increase of the pro-inflammatory cytokine LIGHT in sera of obese children and adolescents. Furthermore, LIGHT levels negatively correlated with Ad-Sos-Z-score, BTT-Z-score, osteocalcin, and 25(OH) vitamin D, but positively correlated with BMI-SDS (the grade of obesity). Our study demonstrates, for the first time, that LIGHT levels are associated with both obesity and bone loss. This finding suggested that LIGHT levels in obese patients could give information on bone status in obese patients. In literature, there is only a study demonstrating the high levels of LIGHT in obese adults [26], which are associated with BMI and altered HOMA-IR. Very interestingly, in this study it is also reported that LIGHT levels decreased following gastric bypass surgery and weight loss. Besides, using in vitro and murine models, different authors try to understand the role of LIGHT in adipose tissue homeostasis, demonstrating either direct or indirect effects of the cytokine, sometimes reaching conflicting results [31,32,33,34]. The high LIGHT levels in the sera of our patients could arose from the high expression of the cytokine on monocytes, CD3^+^-T cells, and neutrophils that we demonstrated by flow cytometry. However, we cannot exclude that the inflamed adipose tissue [32] and liver [43] could represent an additional source of LIGHT in our obese subjects, but ethically we did not have the possibility to justify the use of these samples.

Consistent with our findings, to clarify the mechanisms through which obesity affects bone metabolism, numerous studies have been performed, highlighting the involvement of other pro-inflammatory cytokines. In detail, it has been reported that obesity increases bone resorption by increasing the levels of TNFα and IL-6 [44]. Bone marrow fat also may increase osteoclastogenesis by producing the pro-osteoclastogenic cytokine RANKL [45]. Obese patients displayed low serum levels of adiponectin [46], an inhibitor of osteoclastogenesis [47].

Furthermore, here we also demonstrated LIGHT involvement in the spontaneous osteoclastogenesis, in vitro, in PBMC of obese patients. Consistently, LIGHT is a pro-osteoclastogenic cytokine [21] and high levels have been demonstrated in erosive bone disease, firstly in rheumatoid arthritis [48] and then by bone metastatic non-small cell lung cancer (NSCLC), in osteolytic multiple myeloma, alkaptonuria, chronic kidney disease (CKD), hemodialysis patients, and post-menopausal osteoporosis [19,21,22,23,24,25]. In these different diseases with bone involvement, the elevated LIGHT levels are primarily correlated to its pro-osteoclastogenic role, as we and other authors have reported [21,48]. Previously, in obese children and adolescents, we also demonstrated the high levels of RANKL that support the high osteoclastogenesis in vitro [35]. It has been reported by us and other authors that LIGHT and RANKL displayed a synergic effect on osteoclastogenesis [21,48], thus it is possible that this effect occurred also in obesity. Furthermore, we previously demonstrated that the increased percentage of circulating osteoclast precursors CD14^+^/CD16^+^ in obese children [35] sustained the spontaneous OC formation, also suggesting the possibility their involvement in LIGHT-mediated osteoclastogenesis.

Finally, multiple regression analysis summarized all our findings as it showed that bone and metabolic parameters are the most important predictors of LIGHT levels in obesity.

As a limitation of our studies, we did not have the chance to investigate which receptor is involved in LIGHT-mediated osteoclastogenesis, due to the few milliliters of peripheral blood available from children. However, we previously demonstrated that both transmembrane LIGHT receptors, LTβR and HVEM, are involved in osteoclastogenesis [21], thus we can speculate that they are both involved.

Regarding bone quality assessment in our study, QUS showed several advantages compared with DXA scan, mainly as it is radiation-free. However, although DXA is the gold standard for osteoporosis diagnosis, numerous studies have demonstrated the efficiency of QUS in the screening and the early diagnosis of osteoporosis [49,50,51]. Furthermore, QUS and DXA parameters correlate between them [52,53].

In conclusion, our study highlighted the high serum levels of LIGHT in obese children and adolescents, and its relationship with both the grade of obesity and bone impairment. LIGHT may represent a future therapeutic target to counteract obesity and related comorbidities involving bone disorders.

## 4. Materials and Methods

### 4.1. Patients

We enrolled 111 obese subjects (53 males, mean age 12.21 ± 3.71 years). Among these, 93 attended our outpatient Unit of Pediatric Endocrinology, at University of Bari, and 18 of them were followed at South Paris Specialized Obesity Center, Paris region (Centre Spécialisé Obésité Paris Sud), France. Specialized and integrated centers are the guarantors of optimal management of severe and multi-complicated obesity. The label has been awarded by the Ministry of Health and the Regional Health Agency of Ile-de-France.

Out of them, 64 were obese (Body Mass Index–BMI for age and sex >97th centile), and 47 subjects were affected with severe obesity (BMI >99th centile), according to WHO 2007 guidelines. 

We also enrolled 45 healthy controls (27 males, BMI <85th centile) recruited on a voluntarily basis in the outpatient clinic or among those referred to our hospital for minor surgery or electrocardiographic investigation. The study size was determined considering a power of our tests on LIGHT levels of 0.9. Exclusion criteria were chronic diseases with an impact on bone metabolism (e.g., hypothyroidism or hyperthyroidism, Cushing’s syndrome, celiac disease, or anorexia nervosa), the use of drugs affecting bone turnover (e.g., corticosteroids, antipsychotics, or neuroleptics), and a history of fractures. Written informed consent was obtained from the children’s parents or their legal guardians. All the procedures used were under the guidelines of the Helsinki Declaration on Human Experimentation. 

### 4.2. Anthropometric Measurements

All patients underwent a general clinical examination, anthropometric measurements (height in cm, weight in kg) and data were plotted on Italian or French growth charts and computed as percentiles [54,55]. BMI was defined as weight in kilograms divided by the square of height in meters. The international standards for sex- and age-specific BMI percentiles were used for subjects aged 2–18 years [56]. BMI standard deviation score (SDS) was derived from the published Center for Disease Control and Prevention (CDC) standards [57]. The BMI cut-off point of >2 SDS was used to define obesity, and between 1.4 and 2 SDS to define obesity for individuals <18 years of age. The pubertal and genital stages were assessed according to the Tanner criteria [58]. Both systolic (SBP) and diastolic blood pressure (DBP) were measured in all patients [54], while waist circumference was assessed only in obese subjects [59]. 

### 4.3. Biochemical Measurements

Blood glucose, insulin, total cholesterol (TC), high (HDL) and low (LDL) density lipoprotein cholesterol, triglycerides (TG), and C-reactive protein (CRP) were measured after overnight fasting in all subjects. Values of TC, LDL, HDL, and TG were considered in the normal range if within the 5th and the 95th percentile [60]. Oral glucose (1.75 g/Kg) tolerance test (OGTT) was performed in obese subjects recording basal levels of blood glucose and insulin after 120 min. 

Calcium, phosphorus and alkaline phosphatase (ALP) concentrations were measured by the nephelometric method. Serum active intact parathyroid hormone (PTH) and 25(OH) vitamin D were measured by immunological tests based on the principle of chemiluminescence using commercial kits (Liaison assay; DiaSorin, Stillwater, Minnesota, USA). Osteocalcin serum concentration was measured by enzyme immunoassay (IBL International GmbH, Hamburg, Germany). LIGHT levels were also assessed (R&D Systems, Minneapolis, MN, USA). Two methods to assess insulin resistance, the homeostasis model assessment (HOMA) and the quantitative insulin sensitivity check index (QUICKI) were evaluated.

### 4.4. Quantitative Ultrasonography (QUS)

Bone quality was assessed by QUS measurements, performed with a DBM Sonic 1200 bone profiler (Igea S.r.l., Carpi, MO, Italy) employing a sound frequency of 1.25 MHz. QUS is a radiation-free technique that evaluates bone mineral status in the peripheral skeleton by measuring the amplitude-dependent speed of sound (Ad-SoS) and the bone transmission time (BTT), reflecting the bone properties without the interference of the soft tissue [61]. QUS measurements were performed by the same operator on the second to the fifth proximal phalanges of the non-dominant hand and were expressed as Z-scores calculated based on the normal values for age and sex obtained in a large Italian population sample [61]. 

### 4.5. Flow Cytometry

One-hundred microliters of fresh EDTA peripheral blood were stained with monoclonal conjugated antibody: PE-LIGHT/TNFSF14 (R&D Systems, Minneapolis, MN, USA), FITC-CD3 and FITC-CD14 (BD Pharmigen, San Diego, CA, USA). Flow cytometry acquisition and analysis were performed on a BD Accuri™ C6 flow cytometer (Becton Dickinson Immunocytometry System, Mountain View, CA, USA). Positivity areas were determined using an isotype-matched mAb, and a total of 2000 events for each cell sub-population were acquired.

### 4.6. Osteoclastogenesis

PBMCs were isolated after centrifugation over a density gradient using the Ficoll method. PBMCs were plated in 96-well plates at 4 × 10^5^ cell/well, using Alpha-Minimal Essential Medium (α-MEM, Gibco, Milan, Italy), supplemented with 10% fetal bovine serum, and Antibiotic-Antimycotic (100×, Gibco). To obtain fully differentiated human osteoclasts (OCs), PBMCs were cultured in the presence or absence of a neutralizing anti-LIGHT mAb (R&D Systems Inc., Minneapolis, Minnesota, USA) at 100 ng/mL and 200 ng/mL. At the end of the culture period, cells were fixed and stained for tartrate-resistant acid phosphatase (TRAP—Sigma-Aldrich, Milan, Italy) and OCs were identified as TRAP-positive multinucleated cells with three or more nuclei.

### 4.7. Statistical Analyses

For statistical analysis, the Statistical Package for the Social Sciences (SPSS) for Windows, version 22.0 (SPSS Inc., Chicago, IL, USA) was used. Results are reported as means ± standard deviations (SDs). The Kolmogorov-Smirnov test was used to evaluate the normality of the distribution of all parameters. In parameters with normal distribution, mean values were compared using the unpaired Student t-test, whereas linear correlations were calculated with Pearson’s correlation coefficient. In parameters with skewed distribution, significance was evaluated with the Mann-Whitney test and Spearman’s correlation coefficient, respectively. Lastly, multiple regression analyses were applied to identify the relative strength of each biochemical and clinical variable in predicting LIGHT levels. The limit of statistical significance was set at 0.05.

## Figures and Tables

**Table 1:** Characteristics of study population.

General, Auxological, Biochemical and Bone Quality Data	Controls *N* = 45	Obese Patients *N* = 111
Gender (male/female)	27/18	53/58
Age (yr)	11.04 ± 3.39	12.21 ± 3.71
Tanner Stage (I,II,III,IV,V)	14,14,6,6,5	26, 26, 24, 17, 18
Height SDS	0.18 ± 0.39	0.91 ± 1.13
Weight SDS	0.10 ± 0.25	2.69 ± 0.96 **
BMI-SDS	0.30 ± 0.88	2.67 ± 0.59 **
Waist circumference	57.79 ± 4.58	96.11 ± 12.60 **
Total Cholesterol	161.8 ± 9.10	151.90 ± 43.14
HDL cholesterol	53.24 ± 1.92	48.64 ± 21.68
LDL cholesterol	95.79 ± 5.80	86.87 ± 37.28
Triglycerides mg/dL	61.31 ± 10.52	83.54 ± 48.11
Insulin (microU/mL)	6.58 ± 3.50	21.52 ± 10.85 **
Glucose (mg/dL)	85.83 ± 9.50	111.11 ± 29.17
HOMA-IR	2.36 ± 0.30	4.72 ± 2.93 **
QUICKI	0.38 ± 0.03	0.31 ± 0.04 **
25-OH Vitamin D (ng/mL)	28.64 ± 10.70	24.49 ± 10.43
Osteocalcin (ng/mL)	38.26 ± 19.22	51.02 ± 26.98
B-ALP (U/L)	185 ± 45	214.86 ± 100
PTH (pg/mL)	10.74 ± 0.62	28.99 ± 11.90 *
Calcium (mg/dL)	9.60 ± 0.30	9.80 ± 2.46
Phosphorus (mg/dL)	4.50 ± 1.2	4.69 ± 0.83
Ad-Sos-Z-score	0.68 ± 0.50	−1.35 ± 1.36 *
BTT-Z-score	0.25 ± 0.52	−0.47 ± 1.47 *

SDS: standard deviation score; BMI: body mass index; PTH: parathyroid hormone; B-ALP: bone alkaline phosphatase; QUICKI: quantitative insulin sensitivity check index; * *p* < 0.05; ** *p* < 0.001.

**Table 2:** New: significant correlations for obese patients.

	Weight-SDS	25(OH) Vitamin D	Waist Circumference	Cholesterol	Triglycerides	PTH	Insulin	HOMA-IR	Calcium	Phosphorus	Alkaline Phosphatase	Osteocalcin	Ad-SoS-Z-Score	BTT-Z-Score
Weight-SDS	-	*r* = −0.392, *p* < 0.003;	*r* = 0.607, *p* < 0.0001 *				*r* = 0.091, *p* < 0.01 *	*r* = 0.139, *p* < 0.0001	*r* = −0.145, *p* < 0.0001 *	*r* = −0.196, *p* < 0.0001	*r* = −0.246, *p* < 0.0001		*r* = −0.257, *p* < 0.0001 *	*r* = 0.155, *p* < 0.05 *
BMI-SDS		*r* = 0.322, *p* < 0.01	*r* = 0.593, *p* < 0.0001				*r* = 0.115, *p* < 0.001	*r* = 0.192, *p* < 0.0001	-	*r* = −0.173, *p* < 0.0001	*r* = −0.361, *p* < 0.0001		*r* = −0.405, *p* < 0.0001	*r* = −0.147, *p* < 0.01
HOMA-IR			*r* = 0.384, *p* < 0.0001						*r* = 0.305, *p* < 0.0001	*r* = 0.183, *p* < 0.0001		*r* = 0.261, *p* < 0.0001	*r* = −0.137, *p* < 0.04	*r* = −0.147, *p* < 0.01
QUICKI	*r* = −0.095, *p* < 0.02 *	*r* = 0.111, *p* < 0.005 *	*r* = −0.487, *p* < 0.0001 *	*r* = −0.133, *p* < 0.0001	*r* = −0.367, *p* < 0.0001	*r* = −0.393, *p* < 0.0001			*r* = −0.300, *p* < 0.0001	*r* = −0.306, *p* < 0.0001			*r* = 0.216, *p* < 0.001	*r* = 0.149, *p* < 0.015

* Adjustment for age.

**Table 3:** Multivariate analysis model.

Model 1				0.0001	
	Dependent Variable	Independent Variable	β	*p*	*r*
					0.768
	LIGHT	Tanner stage	0.193	0.002	
		Weight-SDS	0.517	0.0001	
		BMI-SDS	0.249	0.014	
		Cholesterol	0.389	0.0001	
		25 (OH) Vitamin D	−0.749	0.0001	
		Osteocalcin	−0.784	0.0001	
		Ad-SoS-Z-Score	−0.103	0.236	
		BTT-Z-score	−0.871	0.0001	

## References

[1] Molgaard C., Larnkjaer A., Mark A.B., Michaelsen K.F. (2011). Are early growth and nutrition related to bone health in adolescence? The Copenhagen Cohort Study of infant nutrition and growth. Am. J. Clin. Nutr..

[2] Farr J.N., Dimitri P. (2017). The Impact of Fat and Obesity on Bone Microarchitecture and Strength in Children. Calcif. Tissue Int..

[3] Viljakainen H., Ivaska K.K., Paldanius P., Lipsanen-Nyman M., Saukkonen T., Pietilainen K.H., Andersson S., Laitinen K., and Makitie O. (2014). Suppressed bone turnover in obesity: A link to energy metabolism? A case-control study. J. Clin. Endocrinol. Metab..

[4] Reinehr T., de Sousa G., Alexy U., Kersting M., Andler W. (2007). Vitamin D status and parathyroid hormone in obese children before and after weight loss. Eur. J. Endocrinol..

[5] Bini V., Igli Baroncelli G., Papi F., Celi F., Saggese G., Falorni A. (2004). Relationships of serum leptin levels with biochemical markers of bone turnover and with growth factors in normal weight and overweight children. Horm. Res..

[6] Faienza M.F., Santoro N., Lauciello R., Calabro R., Giordani L., Di Salvo G., Ventura A., Delvecchio M., Perrone L., Del Giudice E.M. (2010). IGF2 gene variants and risk of hypertension in obese children and adolescents. Pediatr. Res..

[7] Faienza M.F., Acquafredda A., Tesse R., Luce V., Ventura A., Maggialetti N., Monteduro M., Giordano P., Cavallo L. (2013). Risk factors for subclinical atherosclerosis in diabetic and obese children. Int. J. Med. Sci..

[8] Ciccone M.M., Faienza M.F., Altomare M., Nacci C., Montagnani M., Valente F., Cortese F., Gesualdo M., Zito A., Mancarella R. (2016). Endothelial and Metabolic Function Interactions in Overweight/Obese Children. J. Atheroscler. Thromb..

[9] Miniello V.L., Faienza M.F., Scicchitano P., Cortese F., Gesualdo M., Zito A., Basile M., Recchia P., Leogrande D., Viola D. (2014). Insulin resistance and endothelial function in children and adolescents. Int. J. Cardiol..

[10] Weihrauch-Bluher S., Schwarz P., Klusmann J.H. (2019). Childhood obesity: Increased risk for cardiometabolic disease and cancer in adulthood. Metabolism.

[11] Faienza M.F., Wang D.Q., Fruhbeck G., Garruti G., Portincasa P. (2016). The dangerous link between childhood and adulthood predictors of obesity and metabolic syndrome. Intern. Emerg. Med..

[12] Faienza M.F., Chiarito M., Molina-Molina E., Shanmugam H., Lammert F., Krawczyk M., D’Amato G., Portincasa P. (2020). Childhood obesity, cardiovascular and liver health: A growing epidemic with age. World J. Pediatr..

[13] Hotamisligil G.S. (2006). Inflammation and metabolic disorders. Nature.

[14] Mori G., D’Amelio P., Faccio R., Brunetti G. (2013). The Interplay between the bone and the immune system. Clin. Dev. Immunol..

[15] Lee Y.S., Wollam J., Olefsky J.M. (2018). An Integrated View of Immunometabolism. Cell.

[16] Anandacoomarasamy A., Caterson I., Sambrook P., Fransen M., March L. (2008). The impact of obesity on the musculoskeletal system. Int. J. Obes..

[17] Roy B., Curtis M.E., Fears L.S., Nahashon S.N., Fentress H.M. (2016). Molecular Mechanisms of Obesity-Induced Osteoporosis and Muscle Atrophy. Front. Physiol..

[18] Faienza M.F., D’Amato G., Chiarito M., Colaianni G., Colucci S., Grano M., Corbo F., Brunetti G. (2019). Mechanisms Involved in Childhood Obesity-Related Bone Fragility. Front. Endocrinol..

[19] Brunetti G., Belisario D.C., Bortolotti S., Storlino G., Colaianni G., Faienza M.F., Sanesi L., Alliod V., Buffoni L., Centini E. (2020). LIGHT/TNFSF14 Promotes Osteolytic Bone Metastases in Non-small Cell Lung Cancer Patients. J. Bone Miner. Res..

[20] Brunetti G., Faienza M.F., Colaianni G., Gigante I., Oranger A., Pignataro P., Ingravallo G., Di Benedetto A., Bortolotti S., Di Comite M. (2018). Impairment of Bone Remodeling in LIGHT/TNFSF14-Deficient Mice. J. Bone Miner. Res..

[21] Brunetti G., Rizzi R., Oranger A., Gigante I., Mori G., Taurino G., Mongelli T., Colaianni G., Di Benedetto A., Tamma R. (2014). LIGHT/TNFSF14 increases osteoclastogenesis and decreases osteoblastogenesis in multiple myeloma-bone disease. Oncotarget.

[22] Brunetti G., Rizzi R., Storlino G., Bortolotti S., Colaianni G., Sanesi L., Lippo L., Faienza M.F., Mestice A., Curci P. (2018). LIGHT/TNFSF14 as a New Biomarker of Bone Disease in Multiple Myeloma Patients Experiencing Therapeutic Regimens. Front. Immunol..

[23] Brunetti G., Storlino G., Oranger A., Colaianni G., Faienza M.F., Ingravallo G., Di Comite M., Reseland J.E., Celi M., Tarantino U. (2020). LIGHT/TNFSF14 regulates estrogen deficiency-induced bone loss. J. Pathol..

[24] Brunetti G., Tummolo A., D’Amato G., Gaeta A., Ortolani F., Piacente L., Giordano P., Colucci S., Grano M., Papadia F. (2018). Mechanisms of Enhanced Osteoclastogenesis in Alkaptonuria. Am. J. Pathol..

[25] Cafiero C., Gigante M., Brunetti G., Simone S., Chaoul N., Oranger A., Ranieri E., Colucci S., Pertosa G.B., Grano M. (2018). Inflammation induces osteoclast differentiation from peripheral mononuclear cells in chronic kidney disease patients: Crosstalk between the immune and bone systems. Nephrol. Dial. Transpl..

[26] Dandona P., Ghanim H., Monte S.V., Caruana J.A., Green K., Abuaysheh S., Lohano T., Schentag J., Dhindsa S., Chaudhuri A. (2014). Increase in the mediators of asthma in obesity and obesity with type 2 diabetes: Reduction with weight loss. Obesity.

[27] Sedy J., Bekiaris V., Ware C.F. (2014). Tumor necrosis factor superfamily in innate immunity and inflammation. Cold Spring Harb. Perspect. Biol..

[28] Steinberg M.W., Cheung T.C., Ware C.F. (2011). The signaling networks of the herpesvirus entry mediator (TNFRSF14) in immune regulation. Immunol. Rev..

[29] Ware C.F., Sedy J.R. (2011). TNF Superfamily Networks: Bidirectional and interference pathways of the herpesvirus entry mediator (TNFSF14). Curr. Opin. Immunol..

[30] Tamada K., Shimozaki K., Chapoval A.I., Zhai Y., Su J., Chen S.F., Hsieh S.L., Nagata S., Ni J., Chen L. (2000). LIGHT, a TNF-like molecule, costimulates T cell proliferation and is required for dendritic cell-mediated allogeneic T cell response. J. Immunol..

[31] Tiller G., Laumen H., Fischer-Posovszky P., Finck A., Skurk T., Keuper M., Brinkmann U., Wabitsch M., Link D., Hauner H. (2011). LIGHT (TNFSF14) inhibits adipose differentiation without affecting adipocyte metabolism. Int. J. Obes..

[32] Kim H.M., Jeong C.S., Choi H.S., Kawada T., Yu R. (2011). LIGHT/TNFSF14 enhances adipose tissue inflammatory responses through its interaction with HVEM. FEBS Lett..

[33] Liu C., Ding H., Zhu W., Jiang S., Xu J., Zou G.M. (2013). LIGHT regulates the adipogenic differentiation of mesenchymal stem cells. J. Cell. Biochem..

[34] Kou Y., Liu Q., Liu W., Sun H., Liang M., Kong F., Zhang B., Wei Y., Liu Z., Wang Y. (2019). LIGHT/TNFSF14 signaling attenuates beige fat biogenesis. FASEB J..

[35] Corbo F., Brunetti G., Crupi P., Bortolotti S., Storlino G., Piacente L., Carocci A., Catalano A., Milani G., Colaianni G. (2019). Effects of Sweet Cherry Polyphenols on Enhanced Osteoclastogenesis Associated with Childhood Obesity. Front. Immunol..

[36] Weiler H.A., Janzen L., Green K., Grabowski J., Seshia M.M., Yuen K.C. (2000). Percent body fat and bone mass in healthy Canadian females 10 to 19 years of age. Bone.

[37] Goulding A., Jones I.E., Taylor R.W., Williams S.M., Manning P.J. (2001). Bone mineral density and body composition in boys with distal forearm fractures: A dual-energy x-ray absorptiometry study. J. Pediatr..

[38] Geserick M., Vogel M., Eckelt F., Schlingmann M., Hiemisch A., Baber R., Thiery J., Korner A., Kiess W., Kratzsch J. (2020). Children and adolescents with obesity have reduced serum bone turnover markers and 25-hydroxyvitamin D but increased parathyroid hormone concentrations - Results derived from new pediatric reference ranges. Bone.

[39] Radetti G., Franceschi R., Adami S., Longhi S., Rossini M., Gatti D. (2014). Higher circulating parathormone is associated with smaller and weaker bones in obese children. Calcif. Tissue Int..

[40] Gajewska J., Weker H., Ambroszkiewicz J., Szamotulska K., Chelchowska M., Franek E., Laskowska-Klita T. (2013). Alterations in markers of bone metabolism and adipokines following a 3-month lifestyle intervention induced weight loss in obese prepubertal children. Exp. Clin. Endocrinol. Diabetes Off. J. Ger. Soc. Endocrinol. Ger. Diabetes Assoc..

[41] Dimitri P., Wales J.K., Bishop N. (2011). Adipokines, bone-derived factors and bone turnover in obese children; evidence for altered fat-bone signalling resulting in reduced bone mass. Bone.

[42] Mirzaalian Y., Nourian M., Gholamalizadeh M., Doaei S., Hatami M., Hassanzadeh A., Askari G., Farahi R. (2019). The association of quantitative insulin sensitivity indices (HOMA-IR and QUICKI) with anthropometric and cardiometabolic indicators in adolescents. Arch. Med. Sci. Atheroscler. Dis..

[43] Otterdal K., Haukeland J.W., Yndestad A., Dahl T.B., Holm S., Segers F.M., Gladhaug I.P., Konopski Z., Damas J.K., Halvorsen B. Increased Serum Levels of LIGHT/TNFSF14 in Nonalcoholic Fatty Liver Disease: Possible Role in Hepatic Inflammation. Clin. Transl. Gastroenterol.

[44] Pfeilschifter J., Koditz R., Pfohl M., Schatz H. (2002). Changes in proinflammatory cytokine activity after menopause. Endocr. Rev..

[45] Naveiras O., Nardi V., Wenzel P.L., Hauschka P.V., Fahey F., Daley G.Q. (2009). Bone-marrow adipocytes as negative regulators of the haematopoietic microenvironment. Nature.

[46] Arita Y., Kihara S., Ouchi N., Takahashi M., Maeda K., Miyagawa J., Hotta K., Shimomura I., Nakamura T., Miyaoka K. (2012). Paradoxical decrease of an adipose-specific protein, adiponectin, in obesity. 1999. Biochem. Biophys. Res. Commun..

[47] Oshima K., Nampei A., Matsuda M., Iwaki M., Fukuhara A., Hashimoto J., Yoshikawa H., Shimomura I. (2005). Adiponectin increases bone mass by suppressing osteoclast and activating osteoblast. Biochem. Biophys. Res. Commun..

[48] Edwards J.R., Sun S.G., Locklin R., Shipman C.M., Adamopoulos I.E., Athanasou N.A., Sabokbar A. (2006). LIGHT (TNFSF14), a novel mediator of bone resorption, is elevated in rheumatoid arthritis. Arthritis Rheum..

[49] Cole Z.A., Dennison E.M., Cooper C. (2009). The impact of methods for estimating bone health and the global burden of bone disease. Salud. Publica Mex..

[50] Bauer J.S., Link T.M. (2009). Advances in osteoporosis imaging. Eur. J. Radiol..

[51] Rivas-Ruiz R., Méndez-Sánchez L., Castelán-Martínez O.D., Clark P., Tamayo J., Talavera J.O., Huitrón G., Salmerón-Castro J. (2016). Comparison of International Reference Values for Bone Speed of Sound in Pediatric Populations: Meta-analysis. J. Clin. Densitometr..

[52] Delvecchio M., Soldano L., Lonero A., Ventura A., Giordano P., Cavallo L., Grano M., Brunetti G., Faienza M.F. (2015). Evaluation of impact of steroid replacement treatment on bone health in children with 21-hydroxylase deficiency. Endocrine.

[53] Aceto G., D’Addato O., Messina G., Carbone V., Cavallo L., Brunetti G., Faienza m.F. (2014). Bone health in children and adolescents with steroid-sensitive nephrotic syndrome assessed by DXA and QUS. Pediatr. Nephrol..

[54] Cacciari E., Milani S., Balsamo A., Spada E., Bona G., Cavallo L., Cerutti F., Gargantini L., Greggio N. (2006). Italian cross-sectional growth charts for height, weight and BMI (2 to 20 yr). J. Endocrinol. Investig..

[55] Heude B., Scherdel P., Werner A., Le Guern M., Gelbert N., Walther D., Arnould M., Bellaïche M., Chevallier B., Cheymol J. (2019). A big-data approach to producing descriptive anthropometric references: A feasibility and validation study of paediatric growth charts. Lancet. Digit. Health.

[56] Cole T.J., Bellizzi M.C., Flegal K.M., Dietz W.H. (2000). Establishing a standard definition for child overweight and obesity worldwide: International survey. BMJ.

[57] Kuczmarski R.J., Ogden C.L., Grummer-Strawn L.M., Flegal K.M., Guo S.S., Wei R., Mei Z., Curtin L.R., Roche A.F., Johnson C.L. (2000). CDC growth charts: United States. Adv. Data.

[58] Tanner J.M., Whitehouse R.H. (1976). Clinical longitudinal standards for height, weight, height velocity, weight velocity, and stages of puberty. Arch. Dis. Child..

[59] McCarthy H.D. (2006). Body fat measurements in children as predictors for the metabolic syndrome: Focus on waist circumference. Proc. Nutr. Soc..

[60] Yip P.M., Chan M.K., Nelken J., Lepage N., Brotea G., Adeli K. (2006). Pediatric reference intervals for lipids and apolipoproteins on the VITROS 5,1 FS Chemistry System. Clin. Biochem..

[61] Baroncelli G.I., Federico G., Vignolo M., Valerio G., del Puente A., Maghnie M., Baserga M., Farello G., Saggese G. (2006). Cross-sectional reference data for phalangeal quantitative ultrasound from early childhood to young-adulthood according to gender, age, skeletal growth, and pubertal development. Bone.

